# First evidence of predation on the native endangered Iberian desman (*Galemys pyrenaicus)* and Iberian water shrews (*Neomys anomalus*) by the invasive species American mink using eDNA tools in Extremadura (Spain)

**DOI:** 10.1007/s11033-024-10160-0

**Published:** 2024-12-26

**Authors:** Adriana Ripa, María Jesús Palacios-Gonzalez, José A. Díaz-Caballero, Antonio Espinosa, Francisco Javier Zalba, Juan Luis García-Zapata, José Luis Fernádez-García

**Affiliations:** 1 Genetic and Animal Breeding, Faculty of Veterinary, Universidad de Extremadura, Cáceres, 10071 Spain; 2 Dirección General de Sostenibilidad, Junta de Extremadura, Mérida, 06800 España; 3 Área del Medio Natural, Sociedad de Gestión Pública de Extremadura (GPEX), Junta de Extremadura, Mérida, España; 4 Department of Mathematics, Universidad de Extremadura, Badajoz, 06006 Spain

**Keywords:** Faeces, *Galemys pyrenaicus*, Identification, Invasive species, Predation, *Neovison vison*, *Neomys anomalus*

## Abstract

**Background:**

Wildlife conservation and management aims to restore population declines, it is the vulnerable or endangered populations who require the greatest conservation efforts. In this context, non-invasive sampling has been evaluated as an option for reporting prey/predator impact. *Galemys pyrenaicus* is currently threatened throughout its range, and cohabits with *Nemys anomalus*, in Extremadura (Spain). Predation by American mink and otter has been suggested, but the first one is considered a serious conservation problem. This study has focused on the use of molecular methods based on RT-PCR and DNA sequencing, as it can inform on how invasive predators are harming the desman or the Iberian water shrew, and how these genetic tools can be used to detect predation.

**Methods and results:**

Five samples (faecal and stomach contents) were received and RT-PCR assays were performed using TaqMan probes simultaneously targeting the cytochrome b (cytb) genes of *G. pyrenaicus* and *N. anomalus*, and the presence of both micromammals as prey was assessed. The predator was identified by Sanger sequencing using the nuclear IRBP gene. The assays provide a powerful tool for identification of invasive species, as in this case, but further confirmation by comparative sequence alignment by BLAST search was also necessary.

**Conclusions:**

This study contributes to highlight simultaneously monitor and discriminate predation on specific micromammals with faecal samples of predators. Also supports the use of highly sensitive DNA analysis from samples obtained from predators as an additional methodology to monitor their effects on prey populations.

## Introduction

Conservation and wildlife management aims to reinstate population declines caused by human actions [1], especially for vulnerable or endangered populations, which require the most conservation efforts [2]. Non-invasive sampling has been implemented as the best choice to avoid traditional ‘invasive’ bioprospecting and biomonitoring strategies when studying threatened species [2, 3].

The Iberian desman, *Galemys pyrenaicus* (E. Geoffroy Saint-Hilaire 1811), is a small soricomorph mammal of the Talpidae family, with an endemic distribution in mountainous areas from the middle to the northern of the Iberian Peninsula and in the French Pyrenees. This species is currently threatened in its entire range of distribution [4] and is considered endangered in the Central System Mountains (RD 139/2011: MARM, 2011), especially Extremadura (Spain) [3]. The latest data on the desman are alarming as they show a population decrease [4]. Several reports on Iberian desman have focused on its distribution [5, 6], its and demographic organization [7] as well as on ecology or genetics [8, 9]. However, predation by mesocarnivores such as the American mink *Neovison vison* (Schreber, 1777) [10] and the Eurasian otter *Lutra lutr*a (Linnaeus, 1758) [11] has been suggested to contribute to its extinction [12, 13]. The American mink for fur farms [14]. Currently, this species has become a serious conservation problem in Europe, Asia and South America [15, 16] due to its high predation capacity, particularly on sensitive autochthonous populations of amphibians, micromammals, and seabirds [17, 18]. The interaction between predator and prey have traditionally been studied by morphological analysis of the content of faeces [19]. Non- invasive DNA sampling has recently emerged as a solid alternative for wildlife studies, specially those elusive and difficult to manage species [2]. Furthermore, if we use of molecular methods can help and inform about damage caused by invasive species, and their potential threat to endangered species such as the desman, as well as the effect of predation on other endemic species and their ecological impact. With this aim in mind, two parallel and different molecular methods were used to reveal both, on the one hand, a target-based RT-PCR test to detect limited quantity of genetic material after digestion, and, on the other hand, the Sanger sequencing, method due to its recognized solvency to identify a large number of mesocarnivores using the IRBP gene [20].

## Materials and methods

**Fig. 1:**
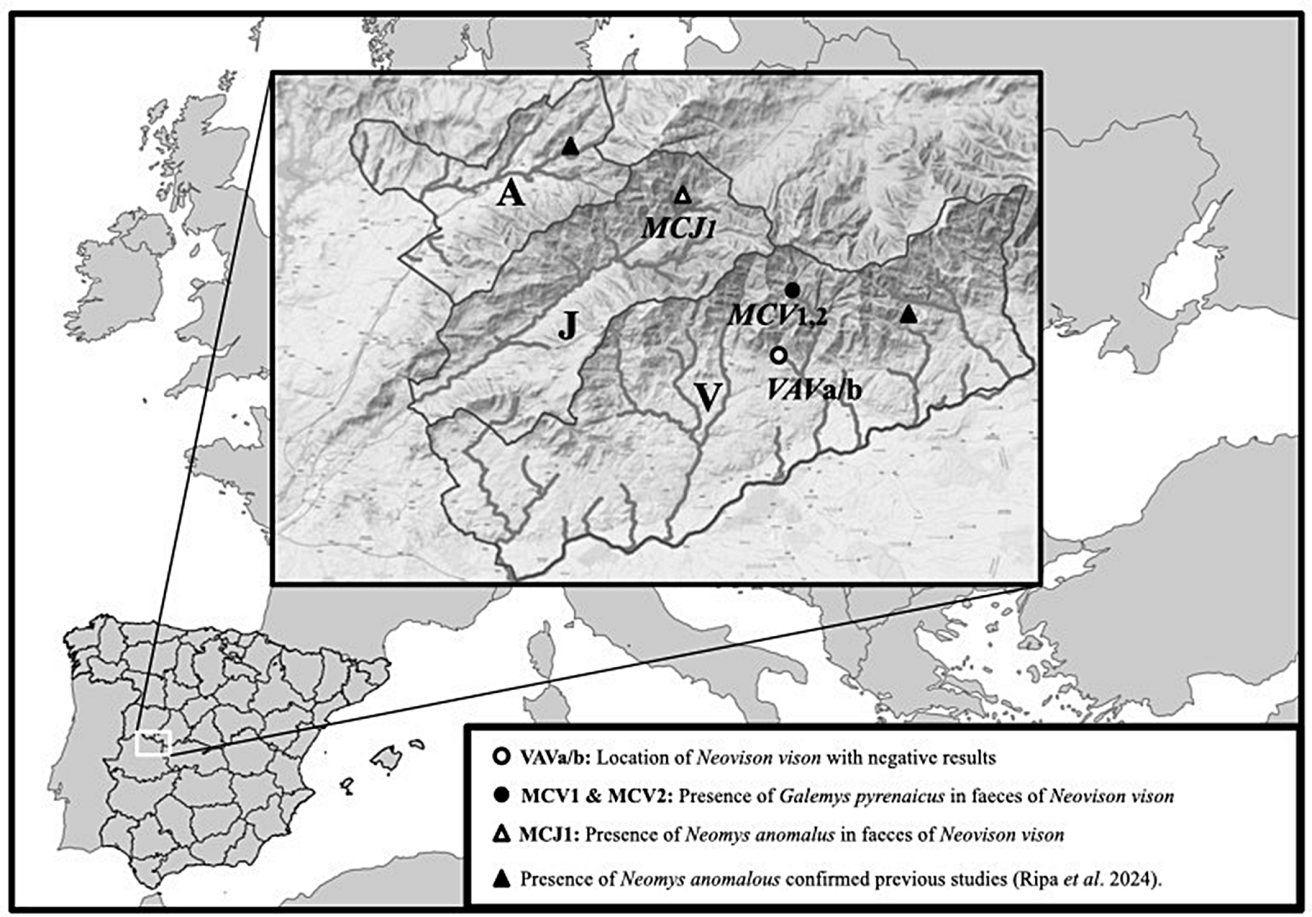
Map showing the three sampling areas with water course, and the location of the samples analyzed: Ambroz (A), Jerte (J), and La Vera (Tietar) (V)

Qualified staff from the regional government of Extremadura were assigned to collect samples (American mink carcasses and faeces of unknown mesocarnivores specimen) between 2019 and 2021 (Fig. 1) and send to our laboratory as a blind sample, only the analytical and laboratory part of the study is carried out in our facilities.

The faecal sampling in three contiguous geographic areas (Valles del Ambroz, del Jerte and del Tiétar (La Vera)) (Fig. 1; Table 1) has been planned and surveyed, due to the fact that recently, populations or individuals of American mink have been detected.

**Table 1:** The table shows the steps that have been followed with the samples. First the identification and collection by the staff of the Junta of Extremadura in correlation with the type of sample that has been analyzed in the laboratory and its subsequent predator and prey identification

ID Samples	Type of sample		Source	Predator identification by IRBP sequencing	Prey identification by RT-PCR assay Galemys/Neomys
(VAVa)	Carcass of mink	Stomach content (a)and faeces (b)	Necropsy	Not required (Phenotypic identification)	-/-
(VAVb)				Not required (Phenotypic identification)	-/-
(MCV1)	Feaces of mink	Faeces content	Enviromental	*Neovison vison*	-/-
(MCV2)	Feaces of mink	Faeces content	Enviromental	*Neovison vison*	-/-
(MCJ1)	Feaces of otter	Faeces content	Enviromental	*Neovison vison*	-/-

The Autonomous Community of Extremadura (Spain), declared that the streams under special monitoring and control should be those with a proven presence of desman (Plan de recuperación del desmán (*Galemys pyrenaicus*) en el Sistema Central (Iberian Peninsula) in Extremadura) (DOE 158 14/08/2018)) [1, 3], and those areas that are under the plan for eradication of invasive species according to (Orden 20 de septiembre de 2018 por la que se aprueba el protocolo para el control y/o erradicación del visón americano (*Neovison vison*) en Extremadura).

In Addition, necropsy samples from a dead American mink from La Vera (VAV1a Stomach content and Faeces VAV1b, Table 1) were included but did not require further molecular identification of the predator, the phenotypic characteristics were enough for analysis. Veterinarian qualified staff were responsible to perform the necropsy and sample collection at their facilities. Field scat and necropsy remains were subsequently transported in sterile tubes containing ethanol (≥ 96%) and stored at − 20 °C during collection in the field and at − 70 °C upon transfer to our laboratory.The rest of the samples, droppings with poor external identification (MCV1, MCV2 MCJ1, Table 1), required molecular confirmation, avoiding confusion between faeces from different semi-aquatic mustelids (e.g. mink, otter, polecat) as they may be species that inhabit the sampling areas.

Double DNA extraction was done for each faeces sample and the stomach content. DNA was isolated using the QIAamp^®^ Fast DNA Stool Mini Kit (QIAGEN GmbH, Hilden, Germany) according to the manufacturer’s instructions in a UV-treated room in a dedicated laboratory. A DNA extraction negative control was always performed on each batch of samples (necropsy stomach contents and faeces). DNA extracts were quantified using a Qubit 4 Fluorometer (Thermo Fisher Scientific, Waltham, MA, USA) and stored at − 70 °C until use.

Micromammal identification was performed by RT-PCR assays using hydrolysis TaqMan probes to detect genetic material from the Iberian desman and the Mediterranean water shrew following Ripa et al. [3]. A presence/absence procedure was performed for each reaction using Design and Analysis Software ver 2.2.1 (Thermo Fisher Scientific-US). The positive cut-off cycle threshold value was considered from Ct ≤ 38 in any case and undetermined to negative cut-off cycle Ct threshold value > 38 [3]. The predator was identified by conventional PCR from faecal remains and subsequent Sanger sequencing of exon 1 of the IRBP nuclear gene as described by Petisco et al. [20]. All assays included a positive control and a negative control. Positive controls for RT-PCR and conventional PCR were a DNA mixture from desman and shrew and one domestic dog and two cats, respectively. The obtained IRBP sequences were compared by BLAST nucleotide search in Gene Bank (NCBI) to download a maximum of 100 additional sequences Then, the phylogenetic tree that relates them was obtained by the “Fast Minimum Evolutionary” distance-based method using the “BLAST tree view” application in NCBI services [21].

## Results and discussion

**Fig. 2:**
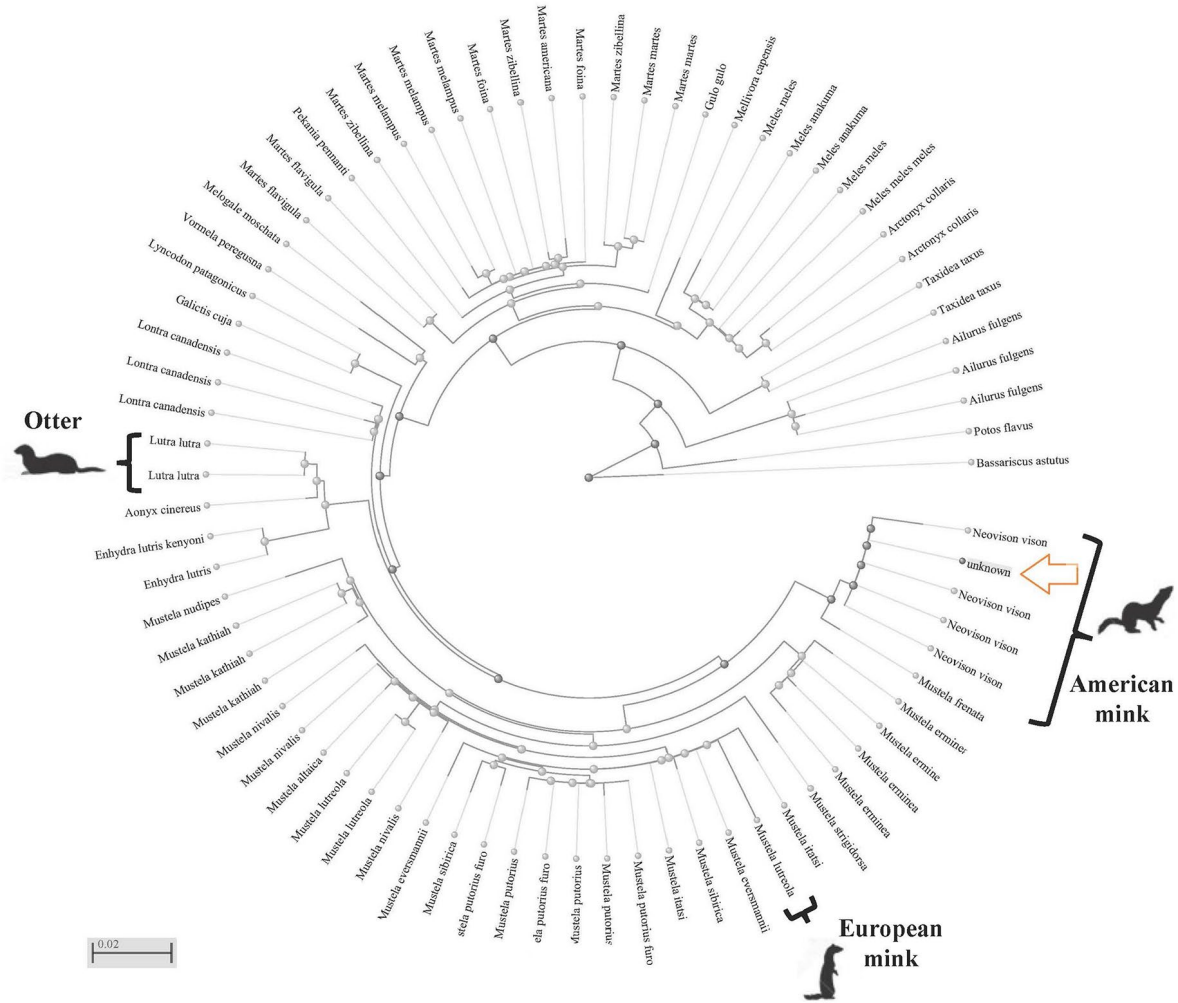
Fast Minimum evolution tree of the sampled IRBP sequence (labeled unknown, indicated with a yellow arrow) and its most similar sequences identified in a BLAST search (identity ≥ 88% molecular weight)

Of the three areas sampled, no remains were found in the Ambroz Valley, however two supposed American mink faeces, in addition to a dead specimen were collected in Tietar (La Vera) and one supposed Otter faeces in Jerte. Also, stomach and faeces samples collected from the American mink carcass neccropsy, but its subsequent analysis did not show predation on the Iberian desman or Mediterranean water shrew (VAV a or b see Table 1). The other two faeces of the MCV1 and MCV2 were analyzed as different samples, but the sender suspected that they belonged to the same predator, as they were collected in close proximity. Positive desman DNA was confirmed on both remains. Subsequent analysis confirmed that both samples belonged to a female desman using the procedure described in Ripa et al. [22]. This provided evidence of predation on this endangered species. Conversely, Mediterranean water shrew predation was detected in the MCJ1 sample in Jerte. These results were compatible with the presence of both prey species in the northern Extremadura, where both micromammals cohabit [3]. As us, remains from faeces of *Lutra lutra* has been analyzed to clarify the prey species using both molecular and morphological techniques, especially relevant if the morphological assignment to species of small feces may be less precise [23]. All IRBP sequences from this study (MCV1, MCV2 and MCJ1) came from American mink species with homozygous genotypes. The sequences were compared with up to a limit of 100 IRBP gene sequences across species available in databases (DDBJ/EMBL/GenBank in the International Nucleotide Sequence Collaboration Databases) at the National Center for Biotechnology Information (NCBI). IRBP gene sequencing clearly assigned all faeces to *Neovison vison*, taking into account that MCJ1 sample was initially suggested by the collector to be from the European otter. The sequences were deposited in the GenBank database (Ac. Nº: PP097362 to PP097364). A BLAST search was performed to compare these IRBP sequences with those in GenBank. The analysis showed a similarity of more than 99% with the American mink captured in Southwest Europe, Central Asia and Japan (respectively Acc. Nº; GQ214074, AB082977, AB119087, respectively) and bioproject PRJNA769404 (Acc. Nº XM_044236829.1), which is why these were clearly attributable to this species. Further taxonomic assignment was be carried out using the BLAST tool. Figure 2 depicted the Fast Minimum Evolution tree using 76 mesocarnivorous sequences with similarity level ≥ 88.

Currently, the analysis of genetic material from non-invasively collected samples, has become a widely used tool in the prospecting and monitoring of wildlife species [24]. Dual DNA assays reveal possible human errors, subjectivity, or inaccuracies during visual research procedures [23], what we also observed in this study (Scat confusion between American mink and Otter). In addition, eDNA metabarcoding are now being used because it provides more detailed dietary information, improving the understanding of resources used by different carnivore species and across multiple habitats [25].

Previous studies [26] have shown that RT-PCR and DNA sequencing can be easily combined for species-specific identification. In fact, genetic identification is highly recommended, especially when morphological analysis identification of prey has limited success [27]. DNA metabarcoding analysis of predators’ diet have shown that digestion slowly breaks down DNA, allowing sufficient intact DNA to be collected [28]. This approach has been used to identify traces present in the digestion of various species. However, species-specific RT-PCR assays proved useful to identify specific prey in research addressing one or few specific targets as in Ripa et al. [3].

According to our results, 1/3 of the remains (assuming that MCV1 and MCV2 are probably to belong to the same individual, based on the collector’s suggestions) in Extremadura were positive for desman predation. This suggests that American mink predation pressure on a critically endangered desman population probably is not negligible. This has already been observed by Romero [29], who found remains of Iberian desman, mandibular bone and dental pieces in 25 out of 95 American mink droppings.

*Lutra lutra* and *Neovison vison* are the main semi-aquatic carnivores declared as possible predators of desman in the Iberian Peninsula [12, 13, 29]. The American mink is the only one of these two carnivorous species that is invasive species, that have been introduced in the 1960s. Today, however, its invasion has prompted plans to eradicate the American mink (RD 630/2013 Spanish Catalogue of Invasive Exotic Species). Semi-aquatic predators inhabit different types of riparian habitats [30, 31], with the mink being the most active along narrow strips of shoreline [32], occupying stable home ranges along riparian sections covered with dense vegetation [30] as well as its very opportunistic diet and much more geared towards mammals than the Otter [33].

Accordingly, because riparian sections covered with dense vegetation also serve as the main site refuges for the desman and the Mediterranean water shrew, they become alley traps when mink are present. In fact, the decline in the range of several riparian mammals, such as the desman, the water vole (*Arvicola sapidus* Miller, 1908) and the Mediterranean water shrew in the mountain rivers of the Central System has been particularity linked to the expansion of the American mink a decade ago [34].

Regarding the presence of desman in the description of the usual diet of the American mink, there are partial data for the Central System [34] because the Extremadura side has not been included to date. However, traces of desman have been found in the faeces of these predators in other samples collected in rivers in Galicia (northeast of Spain). Thus, in habitats where the American mink and the desman coexist simultaneously, the former represents an additional pressure [25] probably incompatible with the survival of the desman in the studied area. Therefore, it is extremely important to fight effectively on a large scale and in a coordinated manner against the American mink, which is damaging a large number of native species [18] with special concern for the desman species. Although morphological identification of fecal remains is useful for ecological research, there are other scenarios in which genetic identification may be more sensitive and effective [24]. In fact, recent studies point to the efficacy and relevance of the application of molecular techniques in those scenarios where ethical and logistical reasons also prevail, particularly claimed in endangered species such as the desman [1].

Finally, this case of the Iberian desman is an excellent example of how a study within the framework of conservation plans may allowed us to assess the impact of native and non-native predators in its range, supported by highly sensitive DNA technologies. Furthermore, our study supports the use of the same genetic material for targeting a prey/predator tandem.

## Data Availability

No datasets were generated or analysed during the current study.
